# Might Banning Trial Publication Do More Harm Than Good?

**DOI:** 10.1371/journal.pmed.0020220

**Published:** 2005-07-26

**Authors:** David Sackett

**Affiliations:** **1**HarlotIrish Lake, OntarioCanada

Smithereens are better than no Smith at all. It was grand to see Richard Smith in full flight again [1], a raptor this time, relegating the randomized controlled trials he previously championed in the *BMJ* to the ether, to be replaced by printed “commentaries.” In doing so, he laid three problematic eggs. First, he shoved systematic reviews and meta-analyses, surely the least biased summaries of efficacy, out of the nest before he took off. Second, the canaries who write commentaries often live in gilded cages provided by the drug industry and printing their pronouncements would make matters worse. Finally, the fledglings who conduct nondrug health-care trials, especially in low- and middle-income countries, shouldn't have their careers stunted by not being able to publish their work in print journals.
